# 2528: A community-academic partnership to understand the correlates of successful aging in place

**DOI:** 10.1017/cts.2017.290

**Published:** 2018-05-10

**Authors:** Rhonda G. Kost, Rhonda G. Kost, Kimberly Vasquez, Dozene Guishard, William Dionne, Caroline Jiang, Cameron Coffran, Andrea Ronning, Glenis George-Alexander, Barry S. Coller, Jonathan N. Tobin

**Affiliations:** Rockefeller University, New York, NY, USA

## Abstract

OBJECTIVES/SPECIFIC AIMS: The Rockefeller University-Center for Clinical and Translational Science and Clinical Directors Network (RU-CCTS/CDN) community-academic-partnership engaged with Carter Burden Center for the Aging (CBCA), a multisite senior community services organization serving Upper Eastside and East Harlem, NY, to develop community-engaged research. Many seniors served by CBCA are racial/ethnic minorities, live in poverty, suffer from multiple chronic conditions, depression, and food insecurity; there is no simple measure routinely used to characterize the health/health risks of program participants. Multiple biological, musculoskeletal, psychosocial and nutritional factors collectively contribute to frailty a construct that is variously defined, and has been used as a surrogate or predictor for health outcomes. Aim 1: We will engage seniors, CBCA leadership, New York City Department for the Aging, staff and other stakeholders in research priority-setting, joint protocol writing, research conduct, analysis and dissemination to cultivate a population of elder stakeholders interested in designing and participating in this and future research. Aim 2: We will characterize the health status of the resident and nonresident populations by collecting data across 3 sessions to include validated cardio-metabolic, musculoskeletal, chronic condition prevalence, quality of life, psychosocial, and nutritional assessments. METHODS/STUDY POPULATION: Stakeholders will be engaged through the process of Community Engaged Research Navigation and a series of meetings and exercises to refine priorities and research design, co-write the protocol, provide feedback on conduct, analyze and disseminate results of the project. RESULTS/ANTICIPATED RESULTS: Outcomes will include rates of participation and retention in assessments and engagement activities, themes from qualitative research, contributions to study design, placement of aims on the T0-T48 spectrum, social network analysis, classification of engagement on the spectrum of Community-based Participatory Research (CBPR) and partnership assessment. The primary outcome is frailty (6-minute walk test); We will examine associations among these measures with services utilization data captured electronically by CBCA. A key deliverable of this project will be a REDCap data capture platform that integrates and displays these measures that will be sustainable for CBCA. DISCUSSION/SIGNIFICANCE OF IMPACT: This practice-based research partnership will allow us to extract, replicate and extend the lessons learned about engaging stakeholders in generating hypotheses, operationalizing research, collecting and analyzing data, and disseminating results. The collaboration is built around generating and testing rigorous clinical an health services hypotheses that are derived from real-world practice-based needs and also incorporate basic science measures to embed and examine mechanistic hypotheses. Testing a simple to implement validated surrogate frailty measure will accelerate progress on evidence-based practices to test interventions that enhance healthy aging and serve as a model for future similar partnerships to form a network for community-based senior research. This work aligns with the RU-CCTS grant Hub Research goal to engage populations across the life span, including hard-to-reach and underserved populations, such as minority seniors.

